# Stress and Anxiety Are Associated with Increased Metabolic Syndrome Risk Among Young Adults Living in the Deep South

**DOI:** 10.3390/healthcare13040359

**Published:** 2025-02-08

**Authors:** Megan E. Renna, Faith N. Wilbourne, Sonal Johal, Ava K. Fergerson, Kylee F. Behringer, Caleb F. Brandner, Jon Stavres, Austin J. Graybeal

**Affiliations:** 1 School of Psychology, University of Southern Mississippi, Hattiesburg, MS 39406, USA; faith.wilbourne@usm.edu (F.N.W.); sonal.johal@usm.edu (S.J.); ava.fergerson@usm.edu (A.K.F.); kylee.behringer@usm.edu (K.F.B.); 2 Department of Kinesiology, Iowa State University, Ames, IA 50011, USA; caleb-brandner@uiowa.edu; 3 School of Kinesiology and Nutrition, University of Southern Mississippi, Hattiesburg, MS 39406, USA; jonathon.stavres@usm.edu (J.S.); austin.graybeal@usm.edu (A.J.G.)

**Keywords:** metabolic syndrome, anxiety, depression, stress, eating behavior, metabolic risk

## Abstract

**Background/Objectives**: This study assessed the association among perceived stress, anxiety, and depression with both the metabolic syndrome (MetS) risk and diagnostic status among young adults in the Deep South. **Methods**: Participants included 132 young adults aged 18–39 (M_age_ = 27.73, SD = 11.11; M_BMI_ = 27.6, SD = 6.8; 56.5% female; 55.7% White) living in Mississippi. In addition to completing self-report measures of perceived stress, anxiety, and depression, all of the participants underwent anthropometric, blood pressure, and fasting blood glucose and lipid assessments to ascertain the MetS status. The participants were provided with both a MetS diagnosis (defined as a dichotomous yes/no variable) as well as a continuous MetS risk severity score determined using existing equations. The risk scores ranged from −1 to +1, with positive scores indicating an increased risk for MetS. **Results**: After controlling for age, biological sex, race, medication use, and education level, multiple regression models revealed significant positive relationships between perceived stress (b = 0.03; *p* = 0.017) and anxiety symptoms (b = 0.01; *p* = 0.039) with the MetS severity. Perceived stress (*p* = 0.017) and anxiety symptoms (*p* = 0.043) were also significantly higher among participants with MetS compared to those without. There were no significant associations between the MetS severity and depressive symptoms, and no differences in depressive symptoms in participants with versus without MetS. **Conclusions**: The results highlight the role of stress and anxiety not only in MetS but in the overall metabolic risk among young adults living in the Deep South. The results highlight the importance of intervening on stress and anxiety early in adulthood to help mitigate cardiometabolic health risk.

## 1. Introduction

An estimated 659,000 American adults die from cardiovascular disease (CVD) annually [1]. In Mississippi, there is an estimated 31% mortality rate from heart attacks and stroke, and over 7900 adults died from heart disease in 2017 [2]. Despite CVD mortality rates decreasing over the past several decades, Mississippi still fares worse in terms of CVD than the majority of the nation overall. In fact, Mississippi has the second highest heart disease mortality rate in the U.S., with 248 deaths per 100,000, and CVD is the leading cause of death for people in Mississippi [3]. Although cardiometabolic risk increases with age, a 2013 study of Mississippians found an increased risk for adults as young as age 35 [4]—highlighting an important need to examine and intervene on CVD risk factors earlier in life to prevent long-term health threats.

Metabolic syndrome (MetS), defined as the accumulation of specific cardiometabolic risk factors, is an established precursor for CVD. Specifically, MetS is diagnosed when someone meets at least three of the following criteria: (1) a waist circumference (WC) ≥ 88 cm for females and ≥102 cm for males; (2) high-density lipoprotein cholesterol (HDL) < 50 mg/dL for females and <40 mg/dL for males; (3) triglycerides (TRGs) ≥ 150 mg/dL; (4) systolic blood pressure (SBP) ≥ 130 mmHg or diastolic blood pressure (DBP) ≥ 85 mmHg; (5) fasting blood glucose (FBG) ≥ 100 mg/dL [5]. In addition to the numerous biological risks of MetS, psychological factors such as anxiety and depression are associated with MetS in middle-aged and older adults [6,7]. Among predominantly middle-aged and older adults, there appears to be a bidirectional association between depressive symptoms and MetS [6], and meta-analytic findings suggest an association between anxiety symptoms and MetS [7]. However, in a large sample of people at risk for CVD, MetS was associated with depressive but not anxiety symptoms among both men and women [8]. A diagnosis of major depression, but not an anxiety disorder, also predicted worse outcomes in terms of treating symptoms associated with MetS [9]. Additionally, people experiencing more symptoms of depression and anxiety are more likely to engage in maladaptive eating patterns in an effort to regulate their negative emotions [10,11], which may inadvertently heighten the cardiometabolic risk through weight gain and increases in glucose, triglycerides, and HDL cholesterol. Taken together, prior research shows a reliable association between both a diagnosis of major depression and depressive symptoms with MetS, but the findings for MetS’ association with anxiety remains ambiguous.

While prior studies have largely focused on examining these associations in middle-aged and older adults, depression and anxiety symptoms are prominent among young adults, who, as previously noted, are experiencing a rapid increase in MetS prevalence [12,13,14]. More broadly, young adults are likely to report high self-reported levels of stress, with high perceived stress serving as a risk factor for decreased quality of life and mental health among these individuals [15,16]. Stress also serves as a risk factor for MetS, with meta-analytic findings showing that people experiencing high stress had a 45% higher chance of being diagnosed with MetS compared to their low-stress counterparts [17]. When assessing different types of stress, this same meta-analysis showed that occupational stress was the highest contributor to MetS risk, while perceived stress was the lowest [17]. Like the findings related to depressive and anxiety symptoms, the mean age of participants across studies were middle-aged, and, to date, it is unknown if the impact of stress on MetS reported in middle-aged and older adults is reflective of its impact among young adults. For example, occupational stress may be less of a central stressor to young adults who are either pursuing higher education or just beginning their careers. Although there is not a robust body of research examining this question, a recent study showed that, among young adults, perceived stress is associated with increased cardiometabolic risk, and adults who had increases in perceived stress from adolescence to young adulthood had the highest level of cardiometabolic risk compared to those with stable or decreasing stress levels [18]. Among college students, higher levels of perceived stress were also associated with higher MetS risk factors, including blood glucose, triglycerides, and blood pressure [19]. Consistent with prior research related to depressive and anxiety symptoms, people who experience higher levels of stress also tend to cope with negative emotions by engaging in binge eating or other maladaptive eating behaviors [10].

This study assessed the relationships between psychological health (e.g., stress, anxiety symptoms, and depressive symptoms), MetS, and MetS risk among young adults living in the Deep South. We hypothesized that stress, depressive symptoms, and anxiety symptoms would be positively associated with a continuous MetS risk score among our young adult participants. Further, we hypothesized that stress, depressive symptoms, and anxiety symptoms would be associated with an increased risk for being diagnosed with MetS, and that these psychological health parameters would be worse among those meeting the criteria for a MetS diagnosis. Given these associations between psychological symptoms and eating behaviors [10], we suspect that emotional eating will impact the strength of the relationship between psychological health and MetS risk. Therefore, as a final exploratory aim, we examined if emotional eating and overeating moderated the relationship between perceived stress, depressive symptoms, or anxiety symptoms with metabolic risk.

## 2. Materials and Methods

### 2.1. Participants and Design

The participants included 132 young adults aged 18–39 (M_age_ = 27.73, SD = 11.11; M_BMI_ = 27.3, SD = 6.8; 56.5% female; 55.7% White) living in the southern region of Mississippi. The inclusion criteria included being between the ages of 18 and 39, and being able to read and understand English. The exclusion criteria included being younger than 18 or older than 39, being pregnant, breastfeeding or lactating, or missing any limbs or part of a limb, or having any portion of a limb casted due to injury. This study was conducted according to the guidelines laid down in the Declaration of Helsinki, and all of the procedures involving human subjects were approved by the university’s Institutional Review Board. Written informed consent was obtained from all subjects.

### 2.2. Procedures

Participants were instructed to arrive at the laboratory after an ≥8 h overnight fast from food, beverages, supplements, and medications, and a ≥24-h abstention from exercise. Upon arrival, and after providing written informed consent, the participants completed a health history and demographic questionnaire, and then had their height measured using a stadiometer, their weight measured using a calibrated digital scale, and their WC and hip circumference measured using a flexible aluminum tape measure. Afterward, the participants sat for ≥5 min prior to the collection of SBP and DBP. Following all anthropometric and blood pressure procedures, capillary blood was collected for the assessment of fasting blood glucose and blood lipids, which were subsequently analyzed using a capillary blood analyzer (Cholestech LDX; Abbott Laboratories, Chicago, IL, USA). Following all of the anthropometric and biological assessments, the participants were escorted to a private room and instructed to complete digital versions (Survey Tool, Qualtrics^®^ LLC, Provo, UT, USA) of the PSS, STAI, CESD, and TFEQ (see below).

### 2.3. Measures

#### 2.3.1. Psychological

The participants completed the 10-item Perceived Stress Scale (PSS) [20] to asses their current stress levels. Scores on the PSS range from 0 to 40, with higher scores indicating more perceived stress over the past month. Cronbach’s α in the current study was 0.85, indicating a good internal consistency. The 20-item State Trait Anxiety Inventory (STAI; trait version) was completed by all of the participants to assess the anxiety symptoms [21]. Scores on the STAI range from 0 to 60, with higher scores indicating more anxiety. Cronbach’s α in the current study was 0.91, indicating excellent internal consistency. The participants also completed the 20-item Center for Epidemiological Studies—Depression (CESD) scale [22]. The CESD is a widely used self-report measure of depressive symptoms, assessed over the past week, with scores ranging from 0 to 60. Higher scores indicate more severe symptoms. Cronbach’s α in the current study was 0.88, indicating good internal consistency.

#### 2.3.2. Dietary

The 18-item Three Factor Eating Questionnaire (TFEQ) assessed typical eating behaviors [23]. Subscales of emotional eating, uncontrolled eating, and restrictive eating were calculated based on participant responses on a 4-point Likert scale for each question. Higher scores on each subscale indicate higher levels of each type of eating behavior. Cronbach’s α for the TFEQ in the current study was 0.86, indicating good internal consistency. Cronbach’s α for each of the subscales in this study ranged from 0.71 to 0.86, indicating acceptable-to-good reliability.

### 2.4. Metabolic Risk

Conventional MetS classification was determined using the guidelines put forth by the Adult Treatment Panel III (ATP-III) [5]. These guidelines include meeting any three out of the following five risk factors: (i) a waist circumference (WC) ≥ 88 cm for females and ≥102 cm for males (for Asian participants, a WC ≥ 80 cm for females and ≥90 cm for males); (ii) high-density lipoprotein cholesterol (HDL) < 50 mg/dL for females and <40 mg/dL for males; (iii) triglycerides (TRGs) ≥ 150 mg/dL; (iv) systolic blood pressure (SBP) ≥ 130 mmHg or diastolic blood pressure (DBP) ≥ 85 mmHg; (v) fasting blood glucose (FBG) ≥ 100 mg/dL.

Recently, a continuous metabolic risk severity score has been used to approximate cardiometabolic risk independent of disease status. This risk score may be particularly beneficial for understanding health among young adults who have not yet developed MetS but are at risk for future health threats. MetS severity (MetS_index_), which provides a continuous *score* representing an individual’s degree of MetS severity across a continuum, as opposed to the conventional classification, was the primary MetS variable, and was calculated using the following equation [24]:

*MetS_index_* = *WCz* + *BPz* − ((*ln*)*HDLz*) + ((*ln*)*TRGz*) + *FBGz*,

where *z* denotes the continuous biomarker value as a standardized z-score. HDL and TRG were log-transformed prior to standardization. The final product of the equation represents the average of the z-scores produced for each variable and is interpreted as such—where a positive score represents an increased MetS severity, a negative score represents a decreased MetS severity, and a score of 0 represents the average severity of the sample.

### 2.5. Statistical Analyses

Post hoc power analyses were conducted for a linear multiple regression and, using a conservative effect size (f = 0.15), it was determined that a total of 98 participants would yield an 80% power at an α = 0.05. All of the analyses were completed in SPSS version 29. Initial analyses explored associations among predictor variables, outcome variables, and covariates to help contextualize associations among the study variables. Hierarchical linear regressions tested the hypothesis that higher anxiety, stress, and depression would be associated with MetS risk. Separate models were run with depressive symptoms, anxiety symptoms, and stress as predictors. All of the models were tested using a two-step approach; covariates were entered into the first step, and depressive symptoms, anxiety symptoms, or stress were entered into the second step to quantify the additional variance explained. Stress, anxiety, and depression levels were also compared between people with and without MetS using independent samples *t*-tests. Moderation models using the Hayes Process [25] (Model 4) tested the hypothesis that eating behavior would moderate the relationship between anxiety symptoms, stress, and depressive symptoms with metabolic risk. Significant interactions were probed at 1 standard deviation above and below the mean of stress, anxiety symptoms, and depressive symptoms. All of the models were adjusted for age, race, ethnicity, medication use, and education level.

## 3. Results

### 3.1. Participant Characteristics

Descriptive statistics are provided in Table 1. Participants had a mean age of 27.73 and were 56.5% female. In terms of race, 55.7% of the participants identified as White, 39.7% as Black, 4.6% as Asian/Pacific Islanders, and 6.9% of Hispanic/Latino. (Although the designation of Hispanic/Latino is not traditionally included or officially recognized in race identification categories according to the U.S. Census, these individuals are often involuntarily lumped into the “other” category. In service of recognizing and appreciating the full range of racial and ethnic identities, we chose to uphold the Hispanic/Latino racial designation in an effort to preserve the strong cultural attachment that some individuals have in identifying their race in this manner (Pew Research Center, 2015), rather than being limited to these census reporting standards.). A total of 20.6% of the participants had MetS. When comparing participants with and without MetS, there were no significant differences in age (M_diff_ = −2.86, t = −1.19; *p* = 0.235). Only 11 participants (8.4%) were taking medications, including birth control, antidepressants, and prescription allergy medications. There were also no differences in groups in terms of sex (*X*^2^(1, N = 131) = 3.43; *p* = 0.06), race (*X*^2^(2, N = 131) = 3.58; *p* = 0.17), or education level (*X*^2^(3, N = 131) = 3.63; *p* = 0.30).

### 3.2. Metabolic Syndrome Risk

When assessing the relationship between psychological variables and the MetS risk score using hierarchical linear regressions, there were significant associations between higher levels of perceived stress and a higher MetS risk (b = 0.03, SE = 0.01; *p* = 0.017). Higher rates of anxiety symptoms were also associated with increased MetS risk (b = 0.01, SE = 0.01; *p* = 0.039). Interestingly, there was no significant association between depressive symptoms and risk for MetS (b = 0.01, SE = 0.01; *p* = 0.242).

### 3.3. Metabolic Syndrome Diagnosis

An independent samples t-test indicated that participants with MetS (M = 42.85, SD = 8.54) scored significantly higher on the STAI than those without MetS (M = 38.73, SD = 9.27) (t(123) = −2.048; *p* = 0.043). Participants with MetS (M = 18.19, SD = 4.91) also scored significantly higher on the PSS when compared to participants without MetS (M = 15.13, SD = 6.10) (t(123) = −2.411; *p* = 0.017). There were no significant differences when comparing the two groups’ scores on the CESD (M_diff_ = −0.64370) (t(125) = −0.357; *p* = 0.722).

### 3.4. Exploratory Analyses—Moderation

Uncontrolled eating was tested as a moderator of the association between perceived stress, anxiety symptoms, and depressive symptoms with metabolic risk. Uncontrolled eating did not moderate the association between perceived stress (B = −0.0003, SE = 0.0007; *p* = 0.64), anxiety symptoms (B = −0.0004, SE = 0.0004; *p* = 0.28), or depressive symptoms (B = −0.0005, SE = 0.0004; *p* = 0.21) and metabolic risk. Similarly, emotional eating did not moderate the relationship between metabolic risk and perceived stress (B = 0.0003, SE = 0.0004; *p* = 0.51), anxiety symptoms (B = 0.0001, SE = 0.0003; *p* = 0.84), or depressive symptoms (B = 0.0002, SE = 0.0003; *p* = 0.51). These non-significant moderation effects were replicated for restrictive eating and perceived stress (B = 0.0003, SE = 0.0005; *p* = 0.51), anxiety symptoms (B = −0.0002, SE = 0.0003; *p* = 0.61), and depressive symptoms (B = 0.0002, SE = 0.0004; *p* = 0.63).

## 4. Discussion

This study assessed the relationship between stress, anxiety symptoms, and depressive symptoms and metabolic risk among young adults living in the Mississippi Delta region, a region at the core of the Deep South. We hypothesized that each psychological predictor would be independently associated with a higher continuous metabolic risk score. Further, we hypothesized that people diagnosed with MetS (approximately 20% of our sample) would report higher levels of stress, depressive symptoms, and anxiety symptoms compared to those without MetS. Our study extends prior research in two important ways. First, most of the past research examining psychological health and MetS has focused on middle-aged and older adults. Additionally, the use of a continuous metabolic risk score expands our understanding of associations between psychological health and cardiometabolic health.

Our results found that higher levels of perceived stress and anxiety symptoms were associated with a higher MetS risk. In contrast to the study hypotheses, depression symptoms were not associated with MetS risk. These contrasting findings between anxiety and depressive symptoms highlight an important distinction when considering the association between psychological symptoms and health risks. Further, these findings were slightly contrary to prior research on associations between depression/anxiety and MetS. While most of the past research highlights an association between depressive symptoms and MetS, the association between MetS and anxiety symptoms is more varied [6,7,8]. One reason for this discrepancy between our findings and prior research is that much of the past work has been in middle-aged and older adults, and has had a greater focus on formal diagnoses of MetS rather than a continuous index of MetS severity. The unique context of the Deep South, where our participants lived, may also highlight one reason for our contrasting depression findings with some prior research. Our findings also extend prior research by showing that higher levels of perceived stress are also associated with an increased MetS risk among the young adults in our sample.

Consistent with study hypotheses, the results showed that people with MetS reported higher levels of perceived stress and anxiety symptoms compared to those without MetS. Interestingly, there were no between-group differences in depressive symptoms. This is in contrast to meta-analytic findings that highlight a bidirectional association between depression and MetS [6]. However, these previous findings were based largely on middle-aged and older adults. The results from this study expand this body of literature by highlighting the associations between psychological health and MetS among young adults—a group that remains understudied in the context of MetS, despite its increasing prevalence of MetS nationwide [26].

People who have higher rates of stress, anxiety symptoms, and depressive symptoms are more likely to engage in binge eating or other maladaptive eating behaviors to try to regulate their negative emotions [10,11]. Given these associations, and eating behaviors’ threat to people’s overall health, we tested the moderating effect of emotional eating on the association between psychological health and MetS risk. In contrast to our hypotheses, entering eating behaviors into our models did not alter the strength or direction of our established associations between stress and anxiety symptoms with the MetS risk, though prior studies have demonstrated that emotional eating is independently associated with MetS severity in young adults [27]. Prior research may benefit from examining other health-promoting and dampening behaviors as potential mediators of the association between mental health and MetS risk.

These findings have important implications for the screening, prevention, and treatment of psychological symptoms. Given the positive associations between perceived stress, anxiety symptoms, and MetS risk, it is possible that, by intervening on these psychological symptoms, mental health providers may effectively be aiding in reducing the MetS risk as well. Further, our results highlight that individuals diagnosed with MetS experience higher levels of anxiety and stress than their healthy counterparts, emphasizing that interventions specifically focused on mental health for people with MetS may prove useful. Several psychological interventions, such as cognitive behavioral therapy or mindfulness- and emotion-focused therapies, should be offered to these individuals to help mitigate some of these psychological risks associated with MetS. Future research should explore whether the delivery of these interventions reduce metabolic risk along with improving mental health.

This study was the first, to our knowledge, to establish associations among psychological health and MetS risk (rather than a formal diagnosis of MetS), offering a more comprehensive picture about how psychological factors such as stress and anxiety may correspond to health risks. Examining stress broadly, and its association with metabolic risk, also provides an important direction for future research to focus on mental health more broadly, rather than specific psychological symptoms and its impact on health risks. Although the rates of metabolic syndrome in this study were somewhat low (20.6%), the rates in this study were reflective of the most recent United States average for the young adult age group (21.3%) [26]. Though MetS diagnosis and severity were assessed using objective measures within the laboratory, several limitations should be considered. First, these data were cross-sectional, and we are therefore unable to test causality in the relationship between psychological health and MetS risk. Further, many of the participants in this study self-reported being moderately or vigorously active (*n* = 105; 64.9%), and the results therefore may not generalize to individuals who live more sedentary lifestyles. Future research would subsequently benefit from examining these associations longitudinally to examine long-term and causal relationships among mental health and metabolic risk. Lastly, although the participants in this study reflected the racial makeup of the geographic region, with a representation of a large sample of both White and Black/African American young adults, future research would benefit from replicating these findings in a more racially and ethnically diverse sample.

## 5. Conclusions

Overall, the results from this study highlight that anxiety symptoms and stress are associated with an increased risk for MetS among young adults living within the Deep South. Additionally, young adults who were diagnosed with MetS endorsed more perceived stress and anxiety symptoms compared to young adults without this diagnosis. Our findings highlight the need for improved screening and interventions at the individual, community, and policy levels for stress and anxiety among young adults to help mitigate some of the known health risks associated with these psychological experiences. These findings also emphasize a benefit for psychoeducation on the health risks associated with stress and anxiety in young adulthood.

## Figures and Tables

**Table 1:** Participant demographics (N = 131).

		Mean (SD)	Number (%)
Age		27.73 (11.10)	-
Gender			
	Male	-	57 (43.5%)
	Female	-	74 (56.5%)
Race			
	White	-	73 (55.7%)
	Black	-	52 (39.7%)
	Asian/Pacific Islander	-	6 (4.6%)
	Hispanic/Latino	-	9 (6.9%)
Physical Activity Level			
	Sedentary	-	9 (6.9%)
	Lightly Active	-	33 (25.2%)
	Moderately Active	-	57 (43.5%)
	Vigorous Activity	-	28 (21.4%)
Using Medication			
	Yes	-	11 (8.4%)
	No	-	120 (91.6%)
BMI		27.30 (6.76)	-
	Normal Weight	-	56 (42.7%)
	Overweight	-	39 (29.8%)
	Obese	-	36 (27.5%)
Systolic Blood Pressure		117.77 (13.51)	
Diastolic Blood Pressure		78.13 (10.18)	
Depressive Symptoms		12.20 (8.29)	
Anxiety Symptoms		39.58 (9.24)	
Perceived Stress		15.77 (5.96)	
MetS Risk Score		−0.23 (0.76)	
MetS Index Classification			
	Negative Score/No MetS	-	84 (64.1%)
	Positive Score/No MetS	-	20 (15.3%)
	Positive Score/MetS	-	27 (20.6%)
Metabolic Syndrome Status			
	No MetS	-	104 (79.4%)
	MetS	-	27 (20.6%)

Note: Anxiety symptoms were assessed via the State Trait Anxiety Inventory. Depressive symptoms were measured via the Center for Epidemiological Studies—Depression Scale. Perceived stress data were collected via the Perceived Stress Scale. Physical activity level was collected via a single-item subjectively rated self-report measure. Medication usage included mental health-related medications, oral contraceptives, and other medications that did not impact metabolic risk factors.

## Data Availability

Data may be made available upon reasonable request to the corresponding author.
